# Case Definitions of Clinical Malaria in Children from Three Health Districts in the North Region of Cameroon

**DOI:** 10.1155/2019/9709013

**Published:** 2019-04-16

**Authors:** Raymond N. Tabue, Boris A. Njeambosay, Francis Zeukeng, Livo F. Esemu, Barrière A. Y. Fodjo, Philomina Nyonglema, Parfait Awono-Ambene, Josiane Etang, Etienne Fondjo, Dorothy Achu, Rose G. F. Leke, Célestin Kouambeng, Tessa B. Knox, Abraham P. Mnzava, Jude D. Bigoga

**Affiliations:** ^1^Ministry of Public Health, National Malaria Control Programme, P.O. Box 14386, Yaoundé, Cameroon; ^2^National Reference Unit for Vector Control, The Biotechnology Center, University of Yaoundé I, P.O. Box 3851-Messa, Yaoundé, Cameroon; ^3^Department of Biochemistry, Faculty of Science, University of Yaoundé I, P.O. Box 812, Yaoundé, Cameroon; ^4^Laboratoire de Recherche sur le Paludisme, Institut de Recherche de Yaoundé, Organisation de Coordination pour la lutte Contre les Endémies en Afrique Centrale, P.O. Box 288, Yaoundé, Cameroon; ^5^Faculty of Medicine and Pharmaceutical Sciences, University of Douala, P.O. Box 2701, Douala, Cameroon; ^6^Institute for Insect Biotechnology, Justus-Liebig-University Gießen, Winchester Str. 2 D-35394 Gießen, Germany; ^7^Global Malaria Programme, World Health Organization, Geneva, Switzerland; ^8^African Leaders Malaria Alliance (ALMA), Dar es Salaam, Tanzania

## Abstract

Malaria endemicity in Cameroon greatly varies according to ecological environment. In such conditions, parasitaemia, which is associated with fever, may not always suffice to define an episode of clinical malaria. The evaluation of malaria control intervention strategies mostly consists of identifying cases of clinical malaria and is crucial to promote better diagnosis for accurate measurement of the impact of the intervention. We sought out to define and quantify clinical malaria cases in children from three health districts in the Northern region of Cameroon. A cohort study of 6,195 children aged between 6 and 120 months was carried out during the raining season (July to October) between 2013 and 2014. Differential diagnosis of clinical malaria was performed using the parasite density and axillary temperature. At recruitment, patients with malaria-related symptoms (fever [axillary temperature ≥ 37.5°C], chills, severe malaise, headache, or vomiting) and a malaria positive blood smear were classified under clinical malaria group. The malaria attributable fraction was calculated using logistic regression models.* Plasmodium falciparum* was responsible for over 91% of infections. Children from Pitoa health district had the highest number of asymptomatic infections (45.60%) compared to those from Garoua and Mayo Oulo. The most suitable cut-off for the association between parasite densities and fever was found among children less than 24 months. Overall, parasite densities that ranged above 3,200 parasites per *μ*l of blood could be used to define the malaria attributable fever cases. In groups of children aged between 24 and 59 months and 60 and 94 months, the optimum cut-off parasite density was 6,400 parasites per *μ*l of blood, while children aged between 95 and 120 months had a cut-off of 800 parasites per *μ*l of blood. In the same ecoepidemiological zone, clinical malaria case definitions are influenced by age and location (health district) and this could be considered when evaluating malaria intervention strategies in endemic areas.

## 1. Introduction

Sub-Saharan African countries remain endemic for malaria and bear the highest morbidity and mortality rates. The risk of having malaria is more pronounced in the Sahelian areas with frequent outbreaks due to seasonality of the transmission. In such areas, individuals, especially children, often harbour malaria parasites, but without clinical symptoms [1].

The policies recommend that in health facilities, malaria should be first diagnosed by the presence of fever and a subsequent confirmatory blood test by microscopy or rapid diagnostic test (RDT). However, this is not always the case due to many challenges such as delay in delivery of certain materials such as RDT or lack of laboratory technician. These weaknesses limit malaria diagnoses to symptoms such as fever only and could contribute to an overdiagnosis of malaria cases and consequently an inappropriate use of antimalarial drugs. Defining clinical malaria cases in such endemic areas is difficult as clinical signs of the disease are nonspecific. Moreover, malaria parasitaemia which is associated with clinical symptoms does not always imply a situation of clinical malaria [2]. Indeed, individuals may carry parasites without symptoms, and coincidental febrile episodes may have etiologies other than malaria. Therefore, it is important to distinguish the disease caused by malaria parasites and the frequent asymptomatic infection caused by the same parasites. This can be done throughout a clear case definition of clinical malaria. The use of a “pyrogenic threshold” has been proposed as the best alternative for defining malaria disease in endemic areas [3, 4]. This variable depends, however, on the transmission intensity, the seasonality of the disease, the patient's immunity, and age. In Mozambique, Smith et al., 1998 [5], reported an age-specific malaria attributable fraction (MAF) among children that decreased with increasing age. This age-specific definition of clinical malaria corroborates with results from other earlier studies in highly endemic areas in sub-Saharan countries [5–7]. MAF is also influenced by immune status. In fact, immunity is linked to transmission intensity and this varies widely in many parts of Africa [8]. Moreover, variations over relatively short distances can still influence clinical definitions of malaria [2]. For instance, in the highlands of western Kenya, Afrane et al., 2014 [9], showed a great variation of MAF among people living in the valley bottom, at the mid-hill and uphill.

A clear and precise approach to estimate the proportion of fever attributable to malaria based on the model of the relationship between fever and parasite density has been reported [1, 10]. This approach also allows the evaluation of the sensitivity and the specificity of the conventional approach in which cases are defined on the basis of fever and specific cut-off values for parasitaemia. We therefore assess the case definitions for clinical malaria in children aged between 6-120 months from three health districts in the savannah areas of the Northern region of Cameroon by using the attributable fraction approach obtained from modelling the relationship between fever risk and parasite density. Specifically, the study aimed to determine the estimated malaria-attributable fever in children by the estimation of the sensitivity and specificity of different parasite density cut-off points.

## 2. Materials and Methods

### 2.1. Study Site

**Figure 1:**
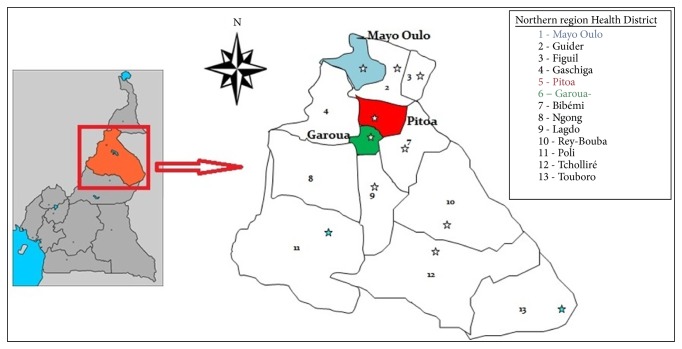
Map of the North Region of Cameroon showing the study health districts (1, 5 and 6).

This study was conducted from 2013 to 2014 during the rainy season in three health districts (HD) in the North Region of Cameroon (Figure 1). Thirty-eight villages were selected, including 12 villages in Pitoa HD (9°23′0′′N, 13° 32′0′′E) located in a rice field area, 17 villages in Garoua HD (9°18′0′′N, 13°24′ 0′′ E) located in periurban and urban areas, and 09 villages in Mayo Oulo HD (9°7′34′′N, 13°37′20′′E) located in the highlands. The choice of selected sites was guided by the existence of available entomological data. These selected HDs are characterized by a Sudanese climate type with an annual rainfall of 700-1000 mm. Malaria transmission here is seasonal with a peak of transmission ranging from September to October.* Plasmodium falciparum *is the most prevalent parasite species and malaria is mainly transmitted by* Anopheles arabiensis, An. gambiae* and* An. funestus *[11]. Other species like* An. pharoensis *and* An. rufipes *play only secondary roles in the transmission of the disease [12, 13]. Agriculture is the prominent activity of the region. Irrigated rice fields and intensive cotton farming characterize Pitoa. In Mayo Oulo, maize and peanuts are mainly cultivated while corn, tomatoes, and eggplant are mainly grown in Garoua.

### 2.2. Active Case Surveillance

A cohort of over 6,195 participants aged between 6-120 months was randomly selected (through a systematic random sampling) from over 1,409 houses in 38 villages. In each health district, houses were randomly selected and all eligible children were enrolled prior to a written consent by their parents or a caregiver. In each village, cohort details of all participants (parent's name, age, sex,…) were gathered. A unique identification was assigned to each participant and corresponded to the household number and the village code. Participants were visited at home, at least once every 2 weeks, and information on the health of enrolled children was recorded and used to identify those who may be having or have experienced fever within the last 48 hours or who they suspected to have malaria. The body axillary temperature was taken with a digital thermometer and the symptoms and signs of the illness were recorded. Every child that was febrile or had been reported to be febrile was tested (TDR) and labelled thin and thick blood smears were prepared. Clinical cases were referred to the nearest hospital for free treatment. A clinical malaria case was defined as an individual with malaria-related symptoms (fever [axillary temperature ≥ 37.5°C], chills, severe malaise, headache, or vomiting) at the time of examination or 1–2 days prior to the examination and the presence of a* Plasmodium* positive blood smear. Cohort members who dropped out were replaced. All cases were entered into the study database monthly. The cohort was followed from 2013 to 2014 during the raining season (July to October). Details of the methods used have previously been described [14].

### 2.3. Laboratory Slide Readings and Plasmodium Species Identification

Thin and thick blood smears were prepared and air-dried; then, the thin smears were fixed in methanol and stained in 4% Giemsa for 30 minutes. Two independent microscopists examined the slides under a ×100 oil immersion microscope objective to obtain a species-specific parasite count. Malaria parasites were counted against 200 white blood cells (WBC) and a blood smear was declared negative after examination of 100 high power fields. The counts were expressed as the number of parasites per microliter of blood, assuming an average leukocyte count of 8,000 cells/ml of blood. For every slide where the difference in parasitaemia between the two readers was greater than five parasites per microliter of blood, a third reader had to re-examine the slide and the mean of the two closest values considered. Free malaria treatment with artesunate-amodiaquine was given to all patients referred to the nearest health centre in case of* Plasmodium* infection as recommended by the national policy.* Plasmodium* species were further confirmed by nested-PCR. Briefly, when a slide was positive for* Plasmodium sp.*, the portion of blood previously spotted on filter papers (Whatman® N°3) was subjected to* Plasmodium* DNA extraction using the Chelex method [15] and the* Plasmodium* species was identified by nested-PCR [16].

### 2.4. Ethical Considerations

The Cameroon National Ethics Committee (102/CNE/SE/09) gave ethical clearance. The children were enrolled into the study if their parents or caregiver provided a written consent after receiving an explanation of the study procedures.

### 2.5. Malaria Case Definitions and Attributable Fraction

The attributable proportion of fever due to malaria was estimated according to the method previously described by Smith [10] and which is based on a logistic regression of the fever on a monotonic function of the parasite density. The logistic regression model used was

(1) $$\begin{array}{c} \log it\left(\;\pi i\;\right)=\alpha +\beta \left(\;xi\;\right)\tau \end{array}$$

Here, (*π*i) represents the probability that an observation (i) with parasite density xi is a fever case. *β*(xi)*τ* corresponds to a type 3 model described by Smith [10] and is a monotonic function which is more flexible than the regression on xi or log* *(xi). The models were fitted using maximum likelihood. To constrain the parameter *τ* to be positive, the maximum likelihood was estimated for the log* *(*τ*). Parasite density cut-off for different study areas and age groups was determined by the intersection of sensitivity and specificity curves. The malaria attributable fraction was calculated as the reduction in the incidence that would be observed if the population was entirely unexposed, compared with its current exposure pattern.

### 2.6. Data Analysis

Data were entered into Epi Info 3.5.3 spread sheet, processed and analysed using the same software. Sampled households were divided according to the health district. Individuals who lived in the same health district were pooled together to calculate the prevalence of asymptomatic malaria infections. The asymptomatic malaria infection rate was calculated as the ratio of infected individuals without symptoms over total samples. The differences in asymptomatic infection rates between districts were determined by chi-square test. Threshold for statistical significance was set at P< 0.05. Parasite thresholds were used to differentiate individuals whose fever was caused by* Plasmodium* from those whose fever had other origin. The sensitivity and specificity of biological and molecular techniques applied for differential diagnosis were used to validate malaria case definition. This was done by an estimation of parasite cut-off densities in different settings, using the point at which the sensitivity and specificity are both highest as the optimum case definition. The prevalence of the disease was used to estimate the fraction of prevalent febrile episodes that are attributable to the presence of malaria in blood.

## 3. Results

### 3.1. Prevalence of Fever and Asymptomatic Malaria Parasite Infections

The prevalence of fever due to malaria in children was higher in Pitoa HD (22.7%) compared to Garoua (18.3%) and Mayo Oulo (15.3%), although the difference was not significant (Chi-squared = 1.48, df = 2, p = 0.48), with an overall prevalence of 19%. The prevalence of fever cases associated with* Plasmodium* infection was 40.95% (Table 1). Among the recorded fever cases associated with* Plasmodium* infection was 33.20% in Garoua, 54.68% in Pitoa, and 30% in Mayo Oulo. The prevalence of fever related to infections varied significantly between investigated health districts (Chi-squared = 9.17, df = 2, p = 0.01).

Children living in Pitoa health district had the highest asymptomatic infection (45.60%) followed by those living in Garoua (24.61%) and Mayo Oulo (15.48%) HDs (Chi-squared = 16.7, df = 2, P = 0.0002). The highest infection rate was found in Pitoa (47.83%) compared to Garoua (26.23%) and Mayo Oulo (17.53%) (Chi-squared = 0.44, df = 2, P = 0.8). Regarding the gametocytes index, this was 12.16%, 11.65%, and 7.30% in Garoua, Mayo Oulo, and Pitoa, respectively (Chi-squared = 120.85, df = 2, P <0.05).* Plasmodium falciparum* was the most prevalent species in the three sites with a percentage of 91.62% and was found in coinfections, with* P. malariae* at very low percentage (2.37). Multiple infections consisted of the association of* P.f+P.m* (5.96%) and* P.f+P.o* (0.05%) (Table 1).

### 3.2. Malaria Parasite Case Definition by Health District

**Figure 2:**
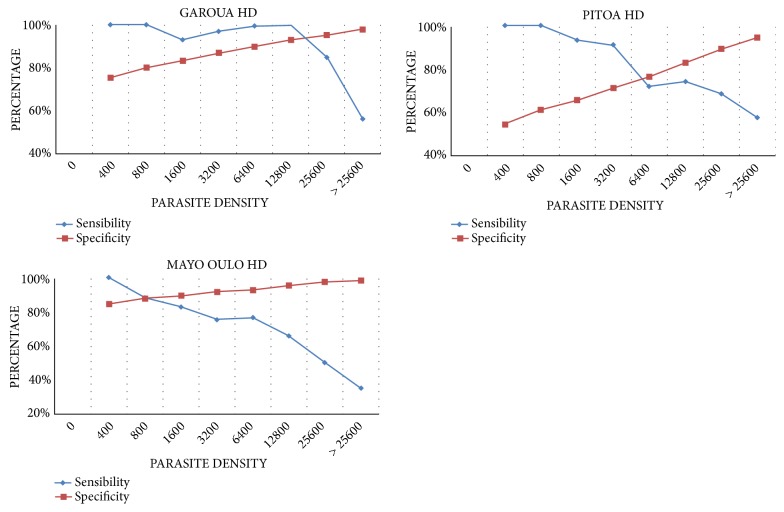
Sensitivity and specificity values at given parasite density for different health districts.

The sensitivity and specificity of various cut-off parasite densities used in the definition of clinical malaria, according to the health district, are given in Figure 2, reproduced from Afrane et al. (2014) [9] [under the Creative Commons Attribution License/public domain].

Overall, the optimum cut-off parasite densities of 800 and 6,400 parasites per *μ*l of blood could be suitable to define malaria attributable cases in children from Mayo Oulo and Pitoa Health districts, respectively. This cut-off was highest at 12,800 parasites per *μ*l of blood in individuals from the Garoua health district.

### 3.3. Malaria Parasite Case Definitions by Age

**Figure 3:**
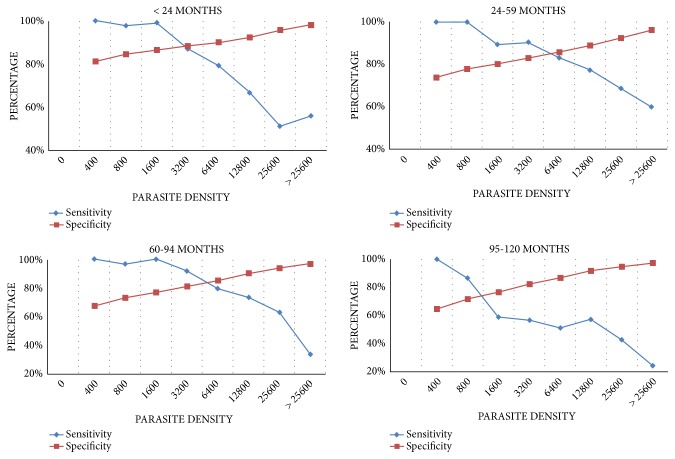
Sensitivity and specificity values at given parasite density for different age groups.

Malaria case definition by age was classified following the optimum cut-off parasite densities (Figure 3). The findings revealed that the parasite density of 3,200 parasites per *μ*l of blood could be used to define the malaria attributable fever cases in children less than 24 months of age. In children aged 24-59 months and 60-94 months, the parasite density of 6,400 parasites per *μ*l of blood is suitable to define the malaria attributable fever cases. This cut-off decreased to 800 parasites per *μ*l of blood in children aged 95–120 months.

### 3.4. Malaria Attributable Fraction

Because the number of fever cases encountered by health district was low, the determination of the malaria attributable fraction (MAF) by age groups in each district was not possible. Thus, cumulative MAFs by age in the three HDs were determined (Figure 3). From the model, the mean malaria-attributable fractions or the probability that any individual fever case was attributable to malaria were 0.11, 0.23, 0.27, and 0.34 in children aged 95-120 months, 60–94 months, 24-59 months, and less than 24 months, respectively. Overall, malaria-attributed fraction decreased with age and varied significantly among investigating health districts with 0.88 in Garoua, 0.49 in Mayo Oulo, and 0.19 in Pitoa (p<0.05), as observed in Table 2, reproduced from Afrane et al. (2014) [9] [under the Creative Commons Attribution License/public domain].

## 4. Discussion

In endemic areas, differential diagnosis of clinical malaria cases is facing many challenges, including the increasing prevalence of asymptomatic malaria infections and the nonspecific symptoms of the disease. Because the evaluation of malaria control interventions is mainly based on the morbidity trend in the population at risk, differential diagnosis is critical for accurate impact intervention measures and for first-line treatment administration [17]. In most health facilities in African rural areas, the lack of equipment and diagnostic reagents for malaria in laboratories as well as qualified laboratory technicians, or the disruption in malaria RDTs supply, remains a serious technical issue to confirm or discard clinical malaria cases. Thus, any fever case is often presumed to be malaria when specific signs of other diseases are lacking [18].

It is well documented that fever symptoms commonly accompany a wide range of childhood illnesses. In general, young children are more vulnerable to infections due to their immature immunity, the different modes of exposure to pathogenic organisms and possibly the low rate of immunisation [19]. Among the 19% fever cases recorded in children, only 40.95% were associated with* Plasmodium* infection. Hence, more than half of febrile children were estimated not to have malaria infection. In the context of limited access to affordable diagnostics and lack of knowledge of the causative agents of other febrile illnesses, most febrile cases are treated and managed as malaria in malaria endemic areas [20]. This inappropriate use of antimalarial drugs may lead to possible development of antimicrobial drug resistance and additional treatment cost due to inappropriate prescriptions. In our study area, most infant fevers may be attributed to opportunistic bacterial or viral infections and might result from an immune system being weakened by malnutrition. Indeed, about 70% of children under five years are highly susceptible to severe acute malnutrition [21].

Our findings revealed that malaria is mesoendemic in investigating study sites, with an overall prevalence of 31.15%. This prevalence did not significantly vary by health district. Although the gametocytemia index was low, the production of gametocytes within an infected human host is essential for transmitting the parasite to the mosquito. In a low transmission context, the proportion of these sexual stages and their density are seminal to understand the transmission of malaria in the population [22]. In the three health districts,* Plasmodium falciparum* was the most predominant malaria parasite species in investigating health districts as previously described [23, 24]. Although* P. vivax* was absent, its presence was reported in the South part of the country [25, 26].

The study revealed a significant difference in MAFs according to health districts. This variation could be due to a difference in the level of transmission as previously described elsewhere [9, 27]. Indeed, malaria transmission in the North Region of Cameroon is spatially and temporally driven by climate variability. In Garoua health district, for instance, agricultural activities performed alongside the Benoue River, as well as paddy fields in Pitoa health district, create breeding sites which are highly prominent for the reproduction of various mosquito species [13]. This heterogeneous environmental disturbance affects the distribution of larval breeding habitats, the spatial distribution of adult vector mosquitoes, and thus could lead to different transmission intensity [13]. As expected, MAFs decreased while the children age increased; this is likely due to the development of immunity.

The differences observed in parasite density cut-off values among children age groups could be attributed to the fact that older people can tolerate higher parasite densities than younger ones. The immunity builds up during many years, preferably after previous exposures and observed episodes could explain this [28, 29]. The parasite density cut-off value to define clinical malaria cases was highest in individuals aged 24-59 months and 60-94 months, thus showing to what extent these children could tolerate malaria parasites in their bodies without getting sick in comparison to children aged less than 24 months. These findings underline how immunity build-up affects asymptomatic parasite carriage. Unexpectedly, the cut-off values dropped to 800 parasites/*μ*l among children aged between 94 and 120 months. Thus, 800 parasites per *μ*l of blood were enough to cause malaria in these children. In contrary, it is well known that older children normally manage high parasite density because of their immune system, which is well developed compared to that of children under five years [27, 30]. The findings suggest a shift in age groups at high risk of clinical episodes of malaria from children under five to older age groups and may be related to the malaria control policy, which is mainly focused on children aged under 5 years and pregnant women. A shift in malaria-affected age groups has also been reported in Sub-Saharan African [31]. This shift was associated with the LLINs coverage. In fact, in some of these countries, the acquisition of funding in 2004 from the Global Fund [32] scaled up malaria control interventions such as insecticide-treated nets (ITNs). It is suggested that focussing on children under 5 years may cause a delay in the acquisition of natural immunity, rendering these children at increased risk of acute infection once they have outgrown the age-specific control interventions at 5 years and older. In the study area like all over the country, LLINs are provided free of charge for children under 5 years and pregnant women [33]. The national policy for malaria treatment recommends free antimalarials with ACT (Artesunate + Amodiaquine) as first-line drug, for children under 5 years and intravenous Artesunate for pregnant women [34]. The country has adopted universal LLINs coverage since 2010, and control interventions such as free treatment strategy remain focused on children under five as well as Seasonal Malaria Chemoprevention (SMC). These results suggest that in Northern Cameroon, there is a considerable shift in the population at risk, from children under five years to older ones. As the cut-off value for parasite density is low in older ages, they could develop severe malaria. Therefore, there is a need to expand prevention strategies such as SMC to school-age children [35, 36] to cover the peak transmission months in order to impact more the disease burden.

However, the study faced some limits such as the fact that we were unable to determine the proportion of patients whose fevers were treated with antipyretic drugs prior to the visit. In fact, fever is the predominant symptom of the use of self-medication by parents for the treatment of their children in Cameroon [37]. This may have influenced the proportion of fever recorded in the study population. Also, the malaria attributable fractions calculated from a single blood-slide do not account for the parasitaemia that may have been undetected by the less sensitive optical microscopy [38]. Furthermore, the density of parasitaemia can fluctuate significantly within hours and therefore use of parasitaemia from a single blood film complicates the modelling [39].

## 5. Conclusion

In the Northern region of Cameroon, where seasonal malaria transmission varies spatially, clinical malaria should be defined by age and by malaria transmission intensity when evaluating interventions. The high prevalence of nonparasitaemic fever underlies the fact that fever is not a specific marker for clinical malaria; hence, there is a need to encourage health workers to perform first-line diagnosis before the administration of an antimalarial treatment. The shift in age groups at high risk of clinical episodes of malaria as observed from children under five to older age groups suggests the need to expand prevention and free treatment strategies for older children in order to cover the peak transmission months.

## Figures and Tables

**Table 1:** Malaria indicators in children from the Northern region of Cameroon.

	Health districts			
Characteristics of malaria infection	Garoua (n=2,666)	Pitoa (n=2,026)	Mayo Oulo (n=1,503)	Total (n=6,195)
Malaria infection prevalence (%)	26.23	47.83	17.53	31.15
Gametocyte rate (%)	12.16	7.3	11.65	9.67
Prevalence of fever (%)	18.3	22.7	15.3	19
Fever + *Plasmodium* infection rate (%)	33.2	54.68	30	40.95
Asymptomatic infection rate (%)	24.61	45.6	15.48	28.9

*Plasmodium species*				
*P. falciparum (P.f) (%)*	93.21	89.2	91.61	91.62
*P. malariae (P.m) (%)*	2.55	2.78	2.37	2.37
*P.f+P.m (%)*	4.24	7.92	3.38	5.96
*P.f+P.o (%)*	0	0.1	0	0.05

n: study population.

**Table 2:** Malaria case definition, parasite density cut-offs, and fever attributable fraction of the cohort in Northern Cameroon.

		Cut-off values (parasites per *μ*l of blood)	n fever	n fever attributable to malaria	malaria-attributable fraction s
Health Districts	Garoua	12,800	18	15.88	0.88
	Pitoa	6,400	23	4.28	0.19
	Mayo Oulo	800	5	2.16	0.43

Age groups (months)	< 24	3,200	4	1.354	0.34
	24-59	6,400	18	4.813	0.27
	60-94	6,400	17	3.949	0.23
	95-120	800	9	0.979	0.11

## Data Availability

The data used to support the findings of this study are available from the corresponding author upon request.
